# Body Mass Index and Vascular Disease in Men Aged 65 Years and Over: HIMS (Health In Men Study)

**DOI:** 10.1161/JAHA.117.007343

**Published:** 2017-11-27

**Authors:** Ben Lacey, Bu B Yeap, Jonathan Golledge, Sarah Lewington, Kieran A McCaul, Paul E Norman, Leon Flicker, Osvaldo P Almeida, Graeme J Hankey

**Affiliations:** ^1^ Western Australian Centre for Health & Ageing Centre for Medical Research University of Western Australia Perth Australia; ^2^ School of Medicine and Pharmacology University of Western Australia Perth Australia; ^3^ Queensland Research Centre for Peripheral Vascular Disease College of Medicine and Dentistry James Cook University Townsville Australia; ^4^ The Department of Vascular and Endovascular Surgery The Townsville Hospital Townsville Australia; ^5^ Department of Endocrinology and Diabetes Fiona Stanley and Fremantle Hospitals Perth Australia; ^6^ Clinical Trial Service Unit and Epidemiological Studies Unit (CTSU) Nuffield Department of Population Health University of Oxford United Kingdom; ^7^ School of Surgery University of Western Australia Perth Australia; ^8^ Department of Geriatric Medicine Royal Perth Hospital Perth Australia; ^9^ School of Psychiatry and Clinical Neurosciences University of Western Australia Perth Australia; ^10^ Department of Neurology Sir Charles Gairdner Hospital Perth Australia; ^11^ MRC Population Health Research Unit Clinical Trial Service Unit and Epidemiological Studies Unit (CTSU) Nuffield Department of Population Health University of Oxford United Kingdom

**Keywords:** adiposity, body mass index, epidemiology, ischemic heart disease, stroke, vascular disease, Epidemiology, Risk Factors, Cardiovascular Disease, Obesity

## Abstract

**Background:**

Understanding the relationship between body mass index (BMI) and vascular disease at older age has become increasingly important in the many countries where both average age and BMI are rising.

**Methods and Results:**

In this prospective cohort study, 12 203 men (aged ≥65) were recruited in 1996–1999 from the general population in Perth, Australia. To limit reverse causality, analyses excluded those with past vascular disease and the first 4 years of follow‐up. During a further 8 (SD3) years of follow‐up, there were 1136 first‐ever major vascular events (nonfatal myocardial infarction, nonfatal stroke, or death from any vascular cause). Cox regression (adjusted for age, education, and smoking) related BMI at recruitment to incidence of major vascular events. At ages 65 to 94, the lowest risk of major vascular events was at ≈ 22.5 to 25 kg/m^2^. In the higher BMI range (≥25 kg/m^2^), 5 kg/m^2^ higher BMI was associated with 33% higher risk of major vascular events (hazard ratio, 1.33 [95% confidence interval, 1.18–1.49]): 24% higher risk of ischemic heart disease (1.24 [1.06–1.46]); 34% higher risk of stroke (1.34 [1.11–1.63]); and 78% higher risk of other vascular death (1.78 [1.32–2.41]). In the lower BMI range, there were fewer events and no strong evidence of an association (hazard ratio per 5 kg/m^2^ higher BMI, 0.82 [95% confidence interval, 0.61–1.12]).

**Conclusions:**

In this population of older men, risk of major vascular events was lowest at ≈ 22.5 to 25 kg/m^2^. Above this range, BMI was strongly related to incidence of major vascular events, with each 5 kg/m^2^ higher BMI associated with ≈30% higher risk.

**Clinical Perspective**

**What Is New?**

- Few prospective studies have assessed the relation of body mass index (BMI) with incidence of vascular disease in older men (≥65 years).
- At ages 65 to 94 years, the lowest risk of major vascular events was at an approximate BMI of 22.5 to 24.9 kg/m^2^.
- Above this range, BMI was strongly related to incidence of major vascular events, with each 5 kg/m^2^ higher BMI associated with around a 30% higher risk; even at ages 85 to 94 years, there was strong evidence of a positive association.
- Around 20% of the excess vascular risk at high BMI was accounted for by the effect of BMI on systolic blood pressure.

**What Are the Clinical Implications?**

- This study quantifies the excess risk of major vascular disease at high BMI in older men.
- It demonstrates that BMI is an important determinant of vascular risk even at very old age.
- In the higher BMI range (>25 kg/m^2^), the proportional differences in vascular risk associated with given difference in BMI were lower than in studies of younger adults, but the absolute difference in risk are greater at older age.

## Introduction

Body mass index (BMI) is a commonly used measure of general adiposity and an established risk factor for vascular disease.^1^, ^2^, ^3^ Despite this, the association between BMI and incidence of major vascular events in older adults has not been well described. Vascular disease is a major cause of disability and death at older age, and understanding its relationship with BMI has become increasingly important to public health, especially in the many countries where both average age and BMI are rising.

The World Health Organization classifies those with BMI <18.5 kg/m^2^ as underweight, 18.5 to 24.9 kg/m^2^ as normal weight, 25.0 to 29.9 kg/m^2^ as overweight, and ≥30.0 kg/m^2^ as obese.^4^ Large studies of mainly middle‐aged adults have reported a J‐shaped association between BMI and major vascular events with the lowest risk in the normal BMI range.^2^, ^3^ The association in older adults (here defined as ≥65 years) is less well established, with some studies indicating that the optimal BMI range may be higher than for middle‐aged adults, whereas others report a more U‐shaped association with increased risk at the extremes of BMI only (ie, underweight or very obese).^5^, ^6^, ^7^

As a result of these findings, it has been suggested that different BMI recommendations are required for older adults at high risk of major vascular events.^7^, ^8^ There have been few large, prospective studies, however, that have specifically addressed this association at older age. We report the findings from a population‐based prospective study of older men in Western Australia. We aimed to: (1) describe the shape and strength of association between BMI and major vascular events in older men; (2) investigate whether the association varies by type of major vascular event; and (3) assess whether other lifestyle risk factors (such as smoking, alcohol intake, and physical activity) are effect modifiers of the association.

## Methods

### Study Design and Participants

For this study, 12 203 men aged 65 to 83 years were recruited from the general population in Perth, Western Australia, in 1996–1999. These men were originally part of a larger, randomized trial of ultrasound screening for abdominal aortic aneurysm, the details of which have been described elsewhere.^9^ In brief, 19 352 men were randomly selected from the electoral roll and invited for screening: 63% attended. Screened men received a letter for their general practitioner that reported the diameter of their abdominal aorta; the letter did not attempt to influence clinical management, and no further interventions were given as part of the trial. At screening, men completed a baseline survey that included questions on sociodemographic factors, lifestyle, and medical history. Also, physical measurements were made, including height (to 0.5 cm), weight (to 0.2 kg), and systolic blood pressure (SBP; to nearest 2 mm Hg). Participants were later invited to a resurvey (2001–2004), which involved a questionnaire and retaking of physical measurements.

Men were monitored following the baseline survey using the Western Australian Data Linkage System to identify deaths and inpatient hospital admissions in Western Australia.^10^ The system has records of admissions from 1970 onward, and codes discharge diagnoses and underlying cause of death to 3 digits using the International Classification of Diseases (ICD) 9 to 10. The primary outcome was first‐ever major vascular event, defined as ischemic heart disease (nonfatal myocardial infarction [ICD‐10: I21–23] or ischemic heart disease death [I20–25]), stroke (nonfatal stroke or stroke death [I60–61, I63–64, H34.1]), or other vascular death (all vascular deaths [I60–99] except stroke or ischemic heart disease; see Table S1 for full list of ICD 9–10 codes).

For this report, we excluded those identified at screening with an enlarged abdominal aorta (≥30 mm in diameter) because these men were likely to have received medical intervention subsequently to address their vascular risk factors (n=875). We also excluded men with a past history of heart disease (n=3419) or stroke (n=1735) to limit the effect of reverse causality; these men were identified by self‐reported history at baseline of ischemic heart disease or stroke, or from discharge diagnoses in the Western Australian Data Linkage System. We further excluded those with missing data on key variables (BMI, age, education, and smoking; n=54). The remaining 7564 men contributed person‐years until first major vascular event (incident cases), death, or the censoring date (December 31, 2010).

Ethics approval for the study was obtained from the Human Research Ethic Committee of the University of Western Australia, and all men provided written informed consent to participate.

### Statistical Analysis

BMI was calculated as the weight of each participant in kilograms divided by the square of their height in meters, as measured at baseline. The hazard ratios (HRs) for the associations between BMI and first major vascular event were calculated using Cox regression models with attained age as the underlying time variable. In categorical analyses, BMI was grouped as follows: 14.0 to 22.4, 22.5 to 24.9, 25.0 to 27.4, 27.5 to 29.9, and 30.0 to 48.0 kg/m^2^. There were too few events in the BMI range <22.5 or ≥30 kg/m^2^ to further divide these groups. Linear associations were produced per 5 kg/m^2^ higher BMI for the BMI ranges <25 and ≥25 kg/m^2^.

The associations were not corrected for regression dilution bias because measures of BMI at baseline and resurvey were strongly correlated. Regression dilution bias results from the inaccuracy (because of technical measurement error or real temporal variation) with which a single measurement of an exposure at baseline characterizes an individual's long‐term average (or “usual”) levels.^11^ The proportional reduction in the strength of the association that results from this bias (the “regression dilution ratio”) can be estimated by comparing baseline and resurvey values. This study used Rosner's regression method to calculate regression dilution ratios,^12^ the ratio being equal to the slope of the regression line between baseline and resurvey BMI values, which were measured, on average, 5.8 years after baseline in 2861 men.

Analyses were adjusted for age at risk, education, and smoking. Assessment of further confounding were made by adjustment for physical activity (self‐reported duration of vigorous and nonvigorous recreational activity was combined into metabolic equivalent‐hours of recreational activity per week), quantity of weekly alcohol intake, region of birth, marital status, and frequency that salt was added to food. Residual reverse causality was assessed by progressively excluding the first 2, 4, and 6 years of follow‐up (ie, this was done to ensure that we did not include participants with pre‐existing disease at baseline that may have affected baseline BMI). Effect modification was investigated by stratifying on age at risk, smoking, alcohol intake, recreational physical activity, and use of blood‐pressure–lowering medication. Both categorical and linear analyses were calculated for BMI versus each type of major vascular event separately (ischemic heart disease, stroke, and other vascular death). We assessed the extent to which the association between BMI and major vascular events was mediated through blood pressure by adjusting the overall association for usual SBP (calculated by dividing the effects of adjustment for baseline SBP by the regression dilution ratio for SBP, as estimated by Rosner's regression method; Table S2).^12^, ^13^

In analyses using categorized BMI, we calculated 95% confidence intervals (CIs) about the HRs using the variance of the log risk,^14^ which appropriately attributes variance to all groups, including the reference, to allow CIs to be used to compare risks in any 2 groups (rather than solely between the reference group and another group). HRs and 95% CIs are presented in the figures so that they illustrate the absolute excess risk at different levels of BMI.^2^, ^14^ This was achieved by multiplying HRs by a common factor so that the inverse‐variance weighted average of the HRs matched the annual incidence of major vascular events in this cohort (Table S3). The proportionality assumption of the Cox models was tested using Schoenfeld residuals and was found to be valid for all of the analyses. Analyses were performed using Stata (v12.0; StataCorp LP, College Station, TX), and figures were plotted using R (v3.0; R Foundation for Statistical Computing, Vienna, Austria).

### Role of Funding Sources

The sponsors of the study had no role in study design, data collection, data analysis, data interpretation, or writing of the report. All authors had full access to all the data in the study and had final responsibility for the decision to submit for publication.

## Results

Following exclusions, there were 7654 men (age 65–84 years) without a past history of cardiovascular disease at baseline. The mean age and BMI of these men at recruitment were 73 (SD, 4) years and 26.7 (SD, 3.7) kg/m^2^, respectively. Several risk factors were strongly associated with BMI at baseline (Table 1). In particular, BMI was negatively associated with education, current smoking, and recreational physical activity and positively associated with several factors (some of which may lie on the causal pathway between BMI and risk of major vascular events), including: diabetes mellitus, SBP, use of blood‐pressure–lowering medication, and use of cholesterol‐lowering medication. There was an approximately linear relation between SBP and BMI: Each 10 kg/m^2^ higher BMI was associated with ≈7 mm Hg higher SBP.

**Table 1:** Characteristics of the 7564 Participants, by Tertile of Baseline BMI

Characteristics	Baseline BMI		
	14 to 25 kg/m^2^	25 to 28 kg/m^2^	28 to 48 kg/m^2^
No. of participants, n	2566	2528	2470
Mean (SD) BMI, kg/m^2^	22.9 (1.8)	26.6 (0.8)	30.8 (2.5)
Mean (SD) age, y	72.1 (4.5)	71.4 (4.2)	71.3 (4.2)
No education beyond primary school, n (%)	451 (17.6)	502 (19.9)	662 (26.8)
Born in Australia, n (%)	1438 (56.0)	1409 (55.7)	1269 (51.4)
Currently married, n (%)	2019 (78.7)	2095 (82.9)	2001 (81.0)
Current smokers, n (%)	405 (15.8)	236 (9.3)	199 (8.1)
Weekly drinkers, n (%)^a^	1605 (65.7)	1692 (70.1)	1662 (69.9)
No recreational physical activity, n %	593 (23.1)	559 (22.1)	724 (29.3)
Mean (SD) MET‐hours of physical activity per week^b^	25.9 (28.8)	26.0 (28.9)	20.2 (24.2)
Self‐reported diabetes mellitus, n (%)	175 (6.8)	216 (8.5)	350 (14.2)
Mean (SD) systolic blood pressure, mm Hg	155.3 (20.8)	158.3 (20.6)	160.9 (20.2)
Always/almost always adds salt to food, n (%)^a^	714 (29.2)	737 (30.6)	780 (32.8)
Using blood‐pressure–lowering medication, n (%)^a^	507 (20.8)	603 (25.0)	876 (36.9)
Using cholesterol‐lowering medication, n (%)^a^	196 (8.0)	232 (9.6)	312 (13.1)

BMI indicates body mass index.

a Information on alcohol intake, frequency that salt is added to food, and medication use was not collected in 332 men.

b MET‐hours=metabolic equivalent hours (a measure which combined the duration and intensity of reported recreational physical activity into a single metric).

Men were resurveyed, on average, 5.8 (SD, 1) years after the baseline survey. There were 2861 men included in the baseline analyses who were resurveyed. BMI values at baseline and resurvey were much the same: using Rosner's regression method, the regression dilution ratio was 0.96, indicating only a minor regression to the mean over this period (Table S4).

At ages 65 to 94 years, there was an approximate J‐shaped association between baseline BMI and incidence of major vascular events (Table S5 and Figure S1). The associations became progressively shallower at the extremes of the BMI range following exclusion of the first 2 and 4 years of follow‐up (further exclusions did not change the strength of these associations). As such, to limit reverse causality, the main prospective analyses reported below exclude the first 4 years of follow‐up. Following these exclusions, there were 1136 major vascular events during a mean follow‐up of 8 (SD, 3) years (mean age at event was 81 [SD, 5] years).

**Figure 1:**
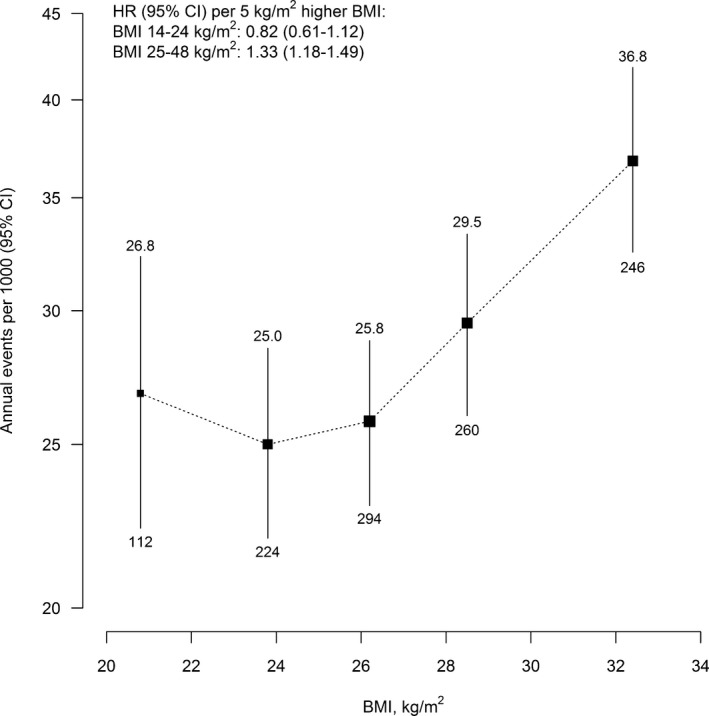
Incidence of major vascular events vs BMI. Hazard ratios (HR) at ages 65 to 94 years for major vascular events vs BMI (excluding the first 4 years of follow‐up and adjusting for age at risk, education, and smoking) were multiplied by a common factor (ie, “floated”) to make the weighted average match the annual incidence of major vascular events in this cohort. Annual incidence was the unweighted average of the component 5‐year incidence rates. For each category, area of square is inversely proportional to the variance of the category‐specific log risk, which also determines the confidence interval. Incidence shown above each square and numbers of events below. BMI indicates body mass index; CI, confidence interval.

Overall, the risk of first major vascular events was lowest in the BMI range 22.5 to 24.9 kg/m^2^ (Figure 1; Table 2). There was strong evidence of a positive association in the higher BMI range (defined as >25 kg/m^2^), with each 5 kg/m^2^ higher BMI associated with an approximate 30% higher risk of major vascular events (HR, 1.33 [95% CI, 1.18–1.49]). In the lower BMI range (<25 kg/m^2^), there were fewer vascular events (especially in the underweight range [n=8]), and there was not strong evidence of an association (HR per 5 kg/m^2^ higher BMI, 0.82 [95% CI, 0.61–1.12]). Analyses were adjusted for age at risk, education, and smoking, and there was no evidence that further adjustment for other potential confounders materially changed the strength of these associations (Table S6).

**Table 2:** Hazard Ratios for Cause‐Specific Incidence of First Major Vascular Events Versus BMI

Baseline BMI, kg/m^2^	Mean BMI, kg/m^2^	Ischemic Heart Disease (594 Events)		Stroke (396 Events)		Other Vascular (146 Events)		All (1136 Events)	
		n	Hazard Ratio (95% CI)	n	Hazard Ratio (95% CI)	n	Hazard Ratio (95% CI)	n	Hazard Ratio (95% CI)
14.0 to 22.4	20.8	52	0.87 (0.66–1.15)	37	1.10 (0.79–1.52)	23	1.97 (1.29–2.99)	112	1.07 (0.89–1.29)
22.5 to 24.9	23.8	126	1.00 (0.84–1.19)	74	1.00 (0.80–1.26)	24	1.00 (0.67–1.49)	224	1.00 (0.88–1.14)
25.0 to 27.4	26.2	166	1.03 (0.88–1.19)	98	1.02 (0.84–1.25)	30	1.02 (0.71–1.46)	294	1.03 (0.92–1.15)
27.5 to 29.9	28.5	135	1.08 (0.91–1.28)	95	1.28 (1.05–1.56)	30	1.37 (0.96–1.97)	260	1.18 (1.04–1.33)
30.0 to 48.0	32.4	115	1.21 (1.00–1.45)	92	1.63 (1.33–2.00)	39	2.41 (1.75–3.31)	246	1.47 (1.30–1.67)

BMI indicates body mass index; CI, confidence interval.

Hazard ratios exclude the first 4 years of follow‐up and adjust for age at risk, education, and smoking.

**Figure 2:**
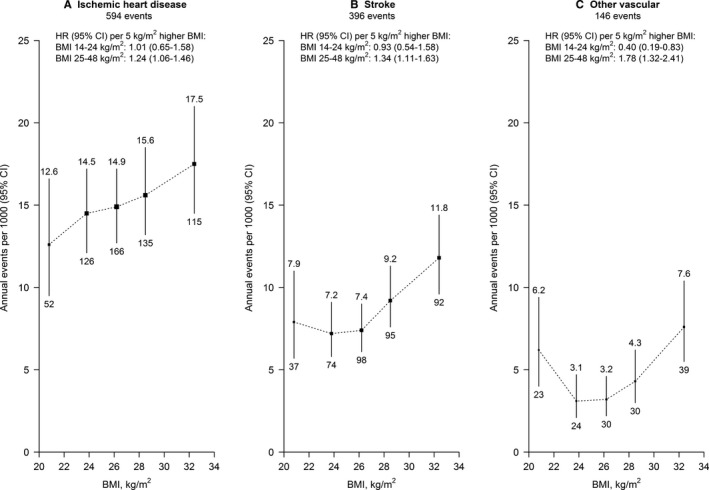
Incidence of cause‐specific major vascular events vs BMI. Hazard ratios (HR) at ages 65 to 94 years for the association of BMI with incidence of (A) ischemic heart disease, (B) stroke, and (C) other vascular disease. Analyses exclude the first 4 years of follow‐up and adjust for age at risk, education, and smoking. HRs were multiplied by a common factor (ie, “floated”) to make the weighted average match the annual incidence of the type of vascular event in this cohort. Annual incidence was the unweighted average of the component 5‐year incidence rates. Risk is indicated on an additive, rather than a multiplicative, scale. For each category, area of square is inversely proportional to the variance of the category‐specific log risk, which also determines the confidence interval. Incidence shown above each square and numbers of events below. BMI indicates body mass index; CI, confidence interval.

The shape and strength of the association varied somewhat by type of major vascular event (Table 2; Figure 2). The association with ischemic heart disease was positive and approximately log‐linear throughout the BMI range examined. The associations with both stroke and other vascular death were also positive in the higher BMI range; but in the lower BMI range, there was no evidence of an association with stroke and an inverse association with other vascular death (Figure 2).

Comparing the strength of the associations in the higher BMI range by type of major vascular event, the association was stronger for other vascular death (HR per 5 kg/m^2^, 1.78 [95% CI, 1.32–2.41]) than for either ischemic heart disease (1.24 [1.06–1.46]) or stroke (1.34 [1.11–1.63]). For analyses by subtype of stroke in this BMI range, there was stronger evidence of an association for ischemic stroke (HR per 5 kg/m^2^, 1.30 [95% CI, 0.99–1.69]; n=197 events) than for intracerebral hemorrhage (0.90 [0.50–1.61]; n=60; Table S7). There was no evidence in this higher BMI range of effect modification of the association for first major vascular events overall by age at risk (65–74, 75–84, and 85–94 years), smoking, alcohol intake, physical activity, and use of blood‐pressure–lowering medication (Table 3). Furthermore, there was no evidence that restricting analyses to men who never smoked regularly (to assess whether the inverse associations at lower BMI could be attributed to smoking) changed the strength of the overall association in the lower BMI range (Figure S2).

**Table 3:** Hazard Ratios for Incidence of First Major Vascular Events vs BMI in the Range 25 to 48 kg/m^2^, by Age at Risk and Baseline Variables

	No. of Events	Mean Age at Event, Y	Hazard Ratio (95% CI)
Age at risk, y			
65 to 74	89	73.1	1.38 (1.03–1.85)
75 to 84	515	80.0	1.29 (1.12–1.48)
85 to 94	196	87.9	1.36 (1.06–1.75)
			Trend, 3 groups: $\chi_{1}^{2}$=0.0 (*P*=1.0)
Smoked regularly			
Never	254	81.5	1.60 (1.30–1.96)
Ever	546	81.0	1.21 (1.05–1.39)
			Heterogeneity: $\chi_{1}^{2}$=4.9 (*P*=0.18)
Alcohol intake^a^			
<Weekly	241	80.9	1.47 (1.22–1.77)
≥Weekly	521	81.3	1.26 (1.08–1.46)
			Heterogeneity: $\chi_{1}^{2}$=1.6 (*P*=0.7)
Recreational physical activity, MET‐hours per week^b^			
0	243	81.3	1.43 (1.19–1.72)
1 to 24	284	81.2	1.21 (0.99–1.48)
≥25	273	81.1	1.28 (1.03–1.59)
			Trend, 3 groups: $\chi_{1}^{2}$=0.6 (*P*=0.9)
Using blood‐pressure–lowering medication			
No	509	81.0	1.34 (1.15–1.55)
Yes	291	81.4	1.22 (1.02–1.47)
			Heterogeneity: $\chi_{1}^{2}$=0.7 (*P*=0.4)
Overall	800	81.2	1.33 (1.18–1.49)

BMI indicates body mass index; CI, confidence interval.

Analyses exclude the first 4 years of follow‐up and adjust for age at risk, education, and smoking.

a Information on alcohol intake was not collected in 332 men.

b MET‐hours=metabolic equivalent hours (a measure which combines duration and intensity of physical activity into a single metric).

**Figure 3:**
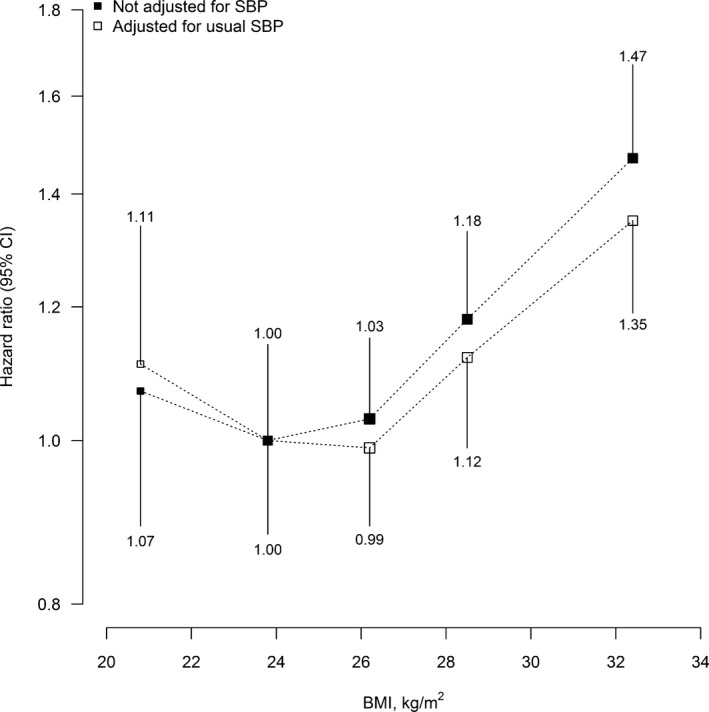
Incidence of major vascular events vs BMI, before and after adjustment for usual SBP. Hazard ratios (HR) at ages 65 to 94 years for major vascular events vs BMI, excluding the first 4 years of follow‐up and adjusting for age at risk, education, and smoking, with further adjustment for usual systolic blood pressure (SBP), where indicated. For each category, area of square is inversely proportional to the variance of the category‐specific log risk, which also determines the confidence interval. HRs are shown above or below each square. BMI indicates body mass index; CI, confidence interval.

Adjusting for usual SBP (regression dilution ratio, 0.42) somewhat attenuated the positive association between BMI and first major vascular events in the higher BMI range (Figure 3). For men with grade 1 obesity (BMI 30–35 kg/m^2^), around 20% of the excess vascular risk from BMI was accounted for by the effect of BMI on SBP.

## Discussion

In this population of older men, the lowest risk of major vascular events was at an approximate BMI of 22.5 to 25 kg/m^2^. There was strong evidence of a positive association in the higher BMI range, with each 5 kg/m^2^ higher BMI associated with around a 30% higher risk of major vascular events. The estimated strength of this association varied somewhat by type of major vascular event, but there was no evidence of effect modification by other lifestyle factors. Around 20% of the excess vascular risk from BMI in this range was accounted for by the effect of BMI on SBP.

This study adds to the limited evidence on the association between BMI and major vascular events at old age. Our findings are broadly consistent with the results of large meta‐analyses on the association of vascular mortality with BMI, conducted in mainly middle‐aged adults.^2^, ^3^, ^15^ The Prospective Studies Collaboration meta‐analysis collated individual participant information from 57 prospective studies with ≈ 30 000 vascular deaths.^2^ The risk of vascular mortality by level of BMI was not reported specifically for older ages in this meta‐analysis, but the shape of the overall association at ages 35 to 79 years (mean, 65 years) between BMI and vascular death was in keeping with the present study. The apparent optimal BMI range to minimize vascular death in this meta‐analysis was 20.0 to 22.5 kg/m^2^ (slightly lower than the present study, but still within the normal BMI range). There were also positive associations for all types of vascular event in the higher BMI range and, in the lower BMI range, a positive association for ischemic heart disease, no association for stroke, and a negative association for other vascular death.

The Prospective Studies Collaboration meta‐analysis also reported the strength of the age‐specific associations in the higher BMI range for ischemic heart disease and subtypes of stroke.^1^ At ages 75 to 84 years, 5 kg/m^2^ higher BMI was associated with 31% (95% CI, 23–39) higher risk of ischemic heart disease, 23% (16–31) higher risk of ischemic stroke, and 32% (22–44) higher risk of intracerebral hemorrhage. These are consistent with the present study for ischemic heart disease and ischemic stroke (there were too few intracerebral hemorrhage events in the present study for statistical stability). Another large meta‐analysis of cohort studies from mainly the Asia‐Pacific region described associations at ages 75 to 84 years that were also consistent with the present study for ischemic heart disease and ischemic stroke, but, unlike the Prospective Studies Collaboration, there was not strong evidence of an association between BMI and intracerebral hemorrhage.^1^, ^3^ The association at ages 75 to 84 years in these meta‐analyses are slightly shallower than those reported in these same meta‐analyses at younger ages. For example, overall (mean age at event, 67 years) in the Prospective Studies Collaboration, each 5 kg/m^2^ higher BMI was associated with 40% higher risk of vascular mortality. Despite this, the absolute differences in risk are likely to be greater at older age.

The associations between BMI and occlusive vascular diseases (ie, ischemic heart disease and ischemic stroke) in the higher BMI range are considered to be largely causal and mediated by the known effects of adiposity on blood pressure, lipids, and diabetes mellitus.^16^ The reason for the apparent shallower association with intracerebral hemorrhage in some studies is also not fully explained, especially given that one of the main mechanisms through which adiposity causes stroke is through its effect on blood pressure, and the prospective association between blood pressure and intracerebral hemorrhage is at least as strong as for ischemic stroke.^17^ It might, however, be explained by the relationships between lipid fractions and stroke subtypes, but these have yet to be quantified reliably for intracerebral hemorrhage.^18^ Furthermore, the lack of positive association at low BMI with some types of vascular event, as shown in the present study, has not been fully explained.

The key strengths of this study include: the objective assessment of height and weight used to measure BMI; the extensive baseline survey which permitted assessment for confounding by a range of vascular risk factors; and the resurvey a few years after the baseline survey, which enabled regression dilution ratios for BMI to be assessed. Furthermore, the Western Australian Data Linkage System allowed the identification of hospitalizations and death from vascular disease throughout the State. Case ascertainment within Western Australia is likely to have been high, and it is estimated that only a small proportion of events will have been missed in men admitted to hospital or dying outside Western Australia.^19^, ^20^, ^21^ The data linkage system also allowed identification of men who had been admitted to hospital in Western Australia for a cardiovascular event preceding the baseline survey, supplementing the self‐reported history of cardiovascular events. The exclusion of these men, together with the first 4 years of follow‐up, will have limited the effect of reverse causality on the associations.

It is a limitation of the study that men were recruited from a trial of screening for aortic aneurysm, given that it is possible that men may have taken steps to address their vascular risk factors following the baseline survey; we excluded those identified as having an abdominal aortic aneurysm for this reason. If men with higher BMI addressed their vascular risk to a greater extent than those of normal body weight (as might be expected given that BMI is correlated with other vascular risk factors), it would have attenuated the associations of major vascular events with BMI. The study would also have benefited from more vascular events to increase the precision of the relative risks, particularly when assessing the effect for rare outcomes, subgroups of interest, or at low BMI (eg, there were only 8 events in the conventional underweight BMI range <18.5 kg/m^2^).^22^

This was a population‐based cohort and therefore more representative of the general population than many highly selected cohorts. However, participants who volunteer for prospective studies are often healthier than the population at large, but it is unlikely that these participants are fundamentally different with respect to the relationships assessed in the present study. A recent representative survey of Western Australian adults reported that around three quarters of men are overweight or obese, even greater than the two thirds in the present analyses.^22^

This study did not assess the effect of central adiposity, or other measures of adiposity, and this should be addressed in future research. Furthermore, it was not possible to assess whether the associations with BMI differ by severity of stroke (such information was not available in the original data set). Further work is also required to assess the relation of BMI with less‐common major vascular events, with minor vascular conditions that do not commonly result in hospitalization, and with other nonvascular causes of death and disability. The combined effects of these conditions with major vascular events will determine the overall apparent ideal BMI range for older men.^23^

Randomized, controlled trials have demonstrated the benefits of weight loss on vascular risk factors, including blood pressure, lipids, and blood glucose concentrations.^16^ This study quantifies the excess vascular risk from high BMI that can be attributable to the effect of BMI on blood pressure, and it is a limitation of the study that bloods were not take at baseline to allow similar analyses for blood glucose or lipid fractions. At an individual level, multiple intervention strategies are often needed to achieve weight loss by targeting dietary change and physical activity, but sometimes pharmacotherapy and surgery are required. Lifestyle interventions for weight loss have been found to be effective in the elderly.^24^ Population‐level approaches to address overweight and obesity require societal change to promote healthier food choices, create environments conducive to regular physical activity, and address weight gain throughout the life course, because it may be easier to avoid weight gain than to lose weight later in life.^25^

In this population of older men, the lowest incidence of major vascular events was at ≈ 22.5 to 25 kg/m^2^ (ie, within the normal BMI range). As such, these findings do not support different recommendations of optimal BMI to address vascular risk in older than younger adults. In the higher BMI range, BMI was strongly associated with all types of major vascular event, with each 5 kg/m^2^ higher BMI associated with around a 30% higher risk of major vascular events overall.

## Sources of Funding

Yeap is recipient of a Clinical Investigator Award from the Sylvia and Charles Viertel Charitable Foundation, New South Wales, Australia. Golledge is supported by grants from the National Health and Medical Research Council, Australia (1079369, 1079193, 1063476, 1022752, 1003707, 1021416, and 1000967), the Queensland Government and the Townsville Hospital Private Practice Trust. Golledge holds a Practitioner Fellowship from the National Health and Medical Research Council, Australia (1019921) and a Senior Clinical Research Fellowship from the Queensland Government. Lewington is funded by the Medical Research Council Population Health Research Unit (MRC PHRU), which is part of the University of Oxford and funded through a strategic partnership between MRC and the University. The Clinical Trial Service Unit and Epidemiological Studies Unit (CTSU) receives core funding from Medical Research Council (UK), British Heart Foundation and Cancer Research UK. The Health In Men Study is supported by grants from the National Health and Medical Research Council, Australia (964145, 139093, 403963, 455811, 1021416, 1000967, 1045710, and 1060557) with additional funding from the National Heart Foundation of Australia, the Western Australian Health Promotion Foundation (Healthway), and Fremantle Hospital Medical Research Foundation. The funding bodies played no role in generation of the data presented in this publication.

## Disclosures

None.

## Supporting information


**Table S1.** Major Vascular Events Endpoints and Their ICD‐9 and ICD‐10 Codes
**Table S2.** Mean SBP at Baseline and Resurvey*
**Table S3.** Number of Major Vascular Events, by Pathological Type and Age at Risk (Among 7564 Participants)
**Table S4.** Baseline and Resurvey BMI, by BMI Baseline Groups (Among 2861 Resurveyed Participants)*
**Table S5.** Hazard Ratios for Incidence of Major Vascular Events Versus BMI, by Period of Follow‐up Excluded
**Table S6.** Incidence of Major Vascular Events Versus BMI, With Progressive Adjustment for Potential Confounders (Excluding the First 4 Years of Follow‐up)
**Table S7.** Hazard Ratios for Incidence of Stroke Subtypes vs BMI (Excluding the First 4 Years of Follow‐up)
**Figure S1.** Incidence of major vascular events vs BMI, without exclusion of the first 4 years of follow‐up.
**Figure S2.** Incidence of major vascular events vs BMI in never smokers only (excluding the first 4 years of follow‐up).
