# Supplementary material for: Body Mass Index and Vascular Disease in Men Aged 65 Years and Over: HIMS (Health In Men Study)
Source: J Am Heart Assoc. 2017 Nov 27;6(12):e007343. doi: 10.1161/JAHA.117.007343 (PMC5779044; doi:10.1161/JAHA.117.007343)
Supplement: Supplementary file 1 — Table S1. Major Vascular Events Endpoints and Their ICD‐9 and ICD‐10 Codes Table S2. Mean SBP at Baseline and Resurvey* Table S3. Number of Major Vascular Events, by Pathological Type and Age at Risk (Among 7564 Participants) Table S4. Baseline and Resurvey BMI, by BMI Baseline Groups (Among 2861 Resurveyed Participants)* Table S5. Hazard Ratios for Incidence of Major Vascular Events Versus BMI, by Period of Follow‐up Excluded Table S6. Incidence of Major Vascular Events Versus BMI, With Progressive Adjustment for Potential Confounders (Excluding the First 4 Years of Follow‐up) Table S7. Hazard Ratios for Incidence of Stroke Subtypes vs BMI (Excluding the First 4 Years of Follow‐up) Figure S1. Incidence of major vascular events vs BMI, without exclusion of the first 4 years of follow‐up. Figure S2. Incidence of major vascular events vs BMI in never smokers only (excluding the first 4 years of follow‐up). [file JAH3-6-e007343-s001.pdf]

Supplemental Material

Table S1. Major vascular events endpoints and their ICD-9 and ICD-10 codes

	ICD-9	ICD-10	Note
Ischaemic heart disease			
Myocardial infarction	410	I21-I23	
Other ischaemic heart disease*	411-414	I20, I24-I25	
Stroke			
Ischaemic stroke	433-434, 362.3	I63, H34.1	Includes central retinal artery occlusion
Intracerebral haemorrhage	431	I61	
Subarachnoid haemorrhage	430	I60	
Unspecified stroke	436	I64	
Other vascular disease*			
Aortic aneurysm	441	I71	
Pulmonary embolism	415	I26	
Heart failure	428	I50	
Hypertensive disease	401-405	I10-I15	Includes hypertension, hypertensive heart disease and hypertensive renal disease
Atherosclerosis & other arterial disease	440, 442-448	I70, I72-I79	Includes peripheral arterial disease, and diseases of arterioles/capillaries
Inflammatory heart disease	420-424	I30-I41	Includes pericarditis, myocarditis, endocarditis
Rheumatic heart disease	390-398	I00-I09	Includes acute and chronic rheumatic heart disease
Other heart disease (not IHD)	416-417,425-427, 429	I27-I28, I42-I49, I51-I52	Includes pulmonary heart disease, cardiomyopathy, dysrhythmia
Other cerebrovascular disease (not stroke)	435, 437-438	I62, I65-69	Includes remainder of cerebrovascular disease ICD-9/10 subchapters not classified as stroke†
Other circulatory disease	451-459	I80-I99	Includes venous disease (including oesophageal varices) and lymphatic disease
All vascular disease	390-459, 362.3	I00-99, H34.1	

* Deaths only (where disease was considered the underlying cause)
† There were no transient ischaemic attack deaths (435 or G45)
n.b. Baseline exclusions: participants with a baseline history of major heart disease or stroke/TIA with the following ICD-9 codes: chronic rheumatic heart disease (393-398); hypertensive heart disease (402,404); pulmonary heart disease (415-416); heart failure (428); ischaemic heart disease (410-414); and stroke/TIA (362.3,430-431,433-436).

Table S2. Mean SBP at baseline and resurvey*

Baseline SBP quintile	Number of participants at baseline	Mean baseline SBP, mmHg	Mean resurvey SBP, mmHg
Quintile 1	627	131.3	135.2
Quintile 2	541	145.8	144.8
Quintile 3	584	155.5	147.3
Quintile 4	548	166.2	152.3
Quintile 5	561	186.4	159.7

*Resurveyed in 2001-2004, on average, 5.8 years after the baseline survey. After excluding participants excluded from the main analysis, there were 2861 resurveyed men.

Table S3. Number of major vascular events, by pathological type and age at risk (among 7564 participants)

Age at risk, years	Person-years at risk	Number of events (rate per 1000 person-years)							
		Ischaemic heart disease		Stroke		Other vascular*		All	
65-74	28775	200	(6.7)	103	(3.5)	13	(0.3)	316	(10.6)
75-84	44279	472	(10.9)	362	(8.5)	102	(2.6)	936	(22.0)
85-94	6615	161	(28.0)	86	(14.0)	58	(11.1)	305	(53.1)
All ages	79669	833	(15.2)	551	(8.7)	173	(4.7)	1557	(28.6)

Rates age-standardised by taking the unweighted average of the component five-year incidence rates.
* Other vascular deaths: 18 aortic aneurysm; 5 pulmonary embolism; 17 heart failure; 30 hypertensive disease; 15 atherosclerosis and other arterial disease; 20 inflammatory heart disease; 1 rheumatic heart disease; 39 other heart disease (not IHD); 21 other cerebrovascular disease (not stroke); 7 other circulatory disease.

Table S4. Baseline and resurvey BMI, by BMI baseline groups (among 2861 resurveyed participants)*

	Number of participants	Mean BMI (SD) at baseline, kg/m²	Mean BMI (SD) at resurvey, kg/m²
**BMI, by quintile at baseline**			
Quintile 1	582	22.4 (1.4)	22.3 (2.2)
Quintile 2	585	24.8 (0.5)	24.7 (1.4)
Quintile 3	572	26.5 (0.5)	26.2 (1.4)
Quintile 4	558	28.1 (0.5)	28.0 (1.6)
Quintile 5	564	31.5 (2.2)	31.1 (3.1)
**BMI, by baseline group, kg/m²**			
14.0-22.4	237	21.0 (1.2)	20.9 (1.7)
22.5-24.9	684	23.9 (0.7)	23.8 (1.8)
25.0-27.4	881	26.2 (0.7)	26.0 (1.5)
27.5-29.9	636	28.5 (0.7)	28.4 (1.9)
30.0-48.0	423	32.1 (2.2)	31.7 (3.1)

*Resurveyed in 2001-2004, on average, 5.8 years after the baseline survey.

Table S5. Hazard ratios for incidence of major vascular events versus BMI, by period of follow-up excluded

Baseline BMI, kg/m^2	Hazard ratio (95% CI), by period of follow-up excluded					
	No years excluded (7564 men, 1557 events)		2 years excluded (7350 men, 1344 events)		4 years excluded (7014 men, 1136 events)	
	n	Hazard ratio (95% CI)	n	Hazard ratio (95% CI)	n	Hazard ratio (95% CI)
14.0-22.4	163	1.15 (0.99-1.35)	137	1.11 (0.94-1.32)	112	1.07 (0.89-1.29)
22.5-24.9	300	1.00 (0.89-1.12)	262	1.00 (0.89-1.13)	224	1.00 (0.88-1.14)
25.0-27.4	393	1.03 (0.93-1.14)	341	1.02 (0.92-1.14)	294	1.03 (0.92-1.15)
27.5-29.9	362	1.26 (1.13-1.39)	306	1.20 (1.07-1.35)	260	1.18 (1.04-1.33)
30.0-48.0	339	1.56 (1.40-1.73)	298	1.55 (1.38-1.73)	246	1.47 (1.30-1.67)

Hazard ratios adjusted for age at risk, education and smoking.

Table S6. Incidence of major vascular events versus BMI, with progressive adjustment for potential confounders (excluding the first 4 years of follow-up)

Baseline BMI, kg/m^2	Number of events	Adjusted for age, education and smoking	Adjusted for age, education, smoking and other factors*	Adjusted for age, education, smoking, physical activity† and other factors*
		Hazard ratio (95% CI)	Hazard ratio (95% CI)	Hazard ratio (95% CI)
14.0-22.4	112	1.07 (0.89-1.29)	1.05 (0.87-1.27)	1.05 (0.87-1.27)
22.5-24.9	224	1.00 (0.88-1.14)	1.00 (0.88-1.14)	1.00 (0.88-1.14)
25.0-27.4	294	1.03 (0.92-1.15)	1.04 (0.93-1.17)	1.04 (0.93-1.17)
27.5-29.9	260	1.18 (1.04-1.33)	1.20 (1.07-1.36)	1.19 (1.06-1.35)
30.0-48.0	246	1.47 (1.30-1.67)	1.47 (1.29-1.67)	1.44 (1.26-1.63)

* Plus place of birth, marital status, frequency salt is added to food and quantity of weekly alcohol intake.
†5 groups by metabolic equivalent hours of recreational [vigorous and non-vigorous] activity per week

Table S7. Hazard ratios for incidence of stroke subtypes versus BMI (excluding the first 4 years of follow-up)

Baseline BMI, kg/m^2	Mean BMI, kg/m^2	Ischaemic stroke (n=197)		Intracerebral haemorrhage (n=60)		Other/unknown (n=139*)	
		n	Hazard ratio (95% CI)	n	Hazard ratio (95% CI)	n	Hazard ratio (95% CI)
14.0-22.4	20.8	14	0.94 (0.55-1.60)	4	0.65 (0.24-1.74)	19	1.51 (0.96-2.38)
22.5-24.9	23.8	33	1.00 (0.71-1.41)	14	1.00 (0.59-1.69)	27	1.00 (0.69-1.46)
25.0-27.4	26.2	51	1.17 (0.89-1.54)	16	0.88 (0.54-1.44)	31	0.91 (0.64-1.29)
27.5-29.9	28.5	50	1.45 (1.10-1.92)	17	1.22 (0.76-1.96)	28	1.09 (0.75-1.58)
30.0-48.0	32.4	49	1.83 (1.38-2.43)	9	0.86 (0.45-1.66)	34	1.79 (1.27-2.51)

Hazard ratios adjust for age at risk, education and smoking.
*7 events were subarachnoid haemorrhage and 132 events were of unknown subtype.

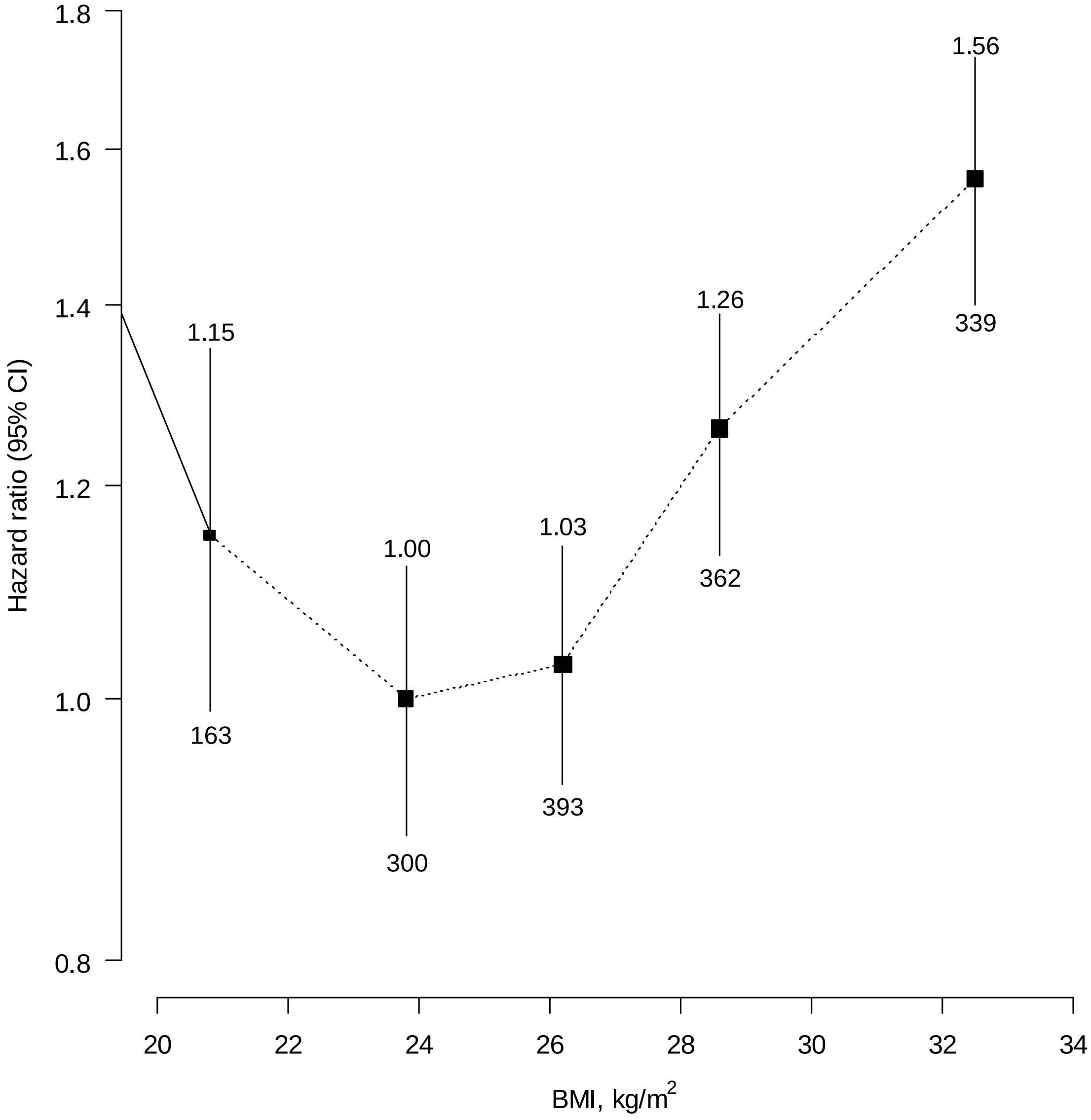

Figure S1. Incidence of major vascular events versus BMI, without exclusion of the first 4 years of follow-up

Hazard ratios adjust for age at risk, education and smoking. Conventions as per Figure 1.

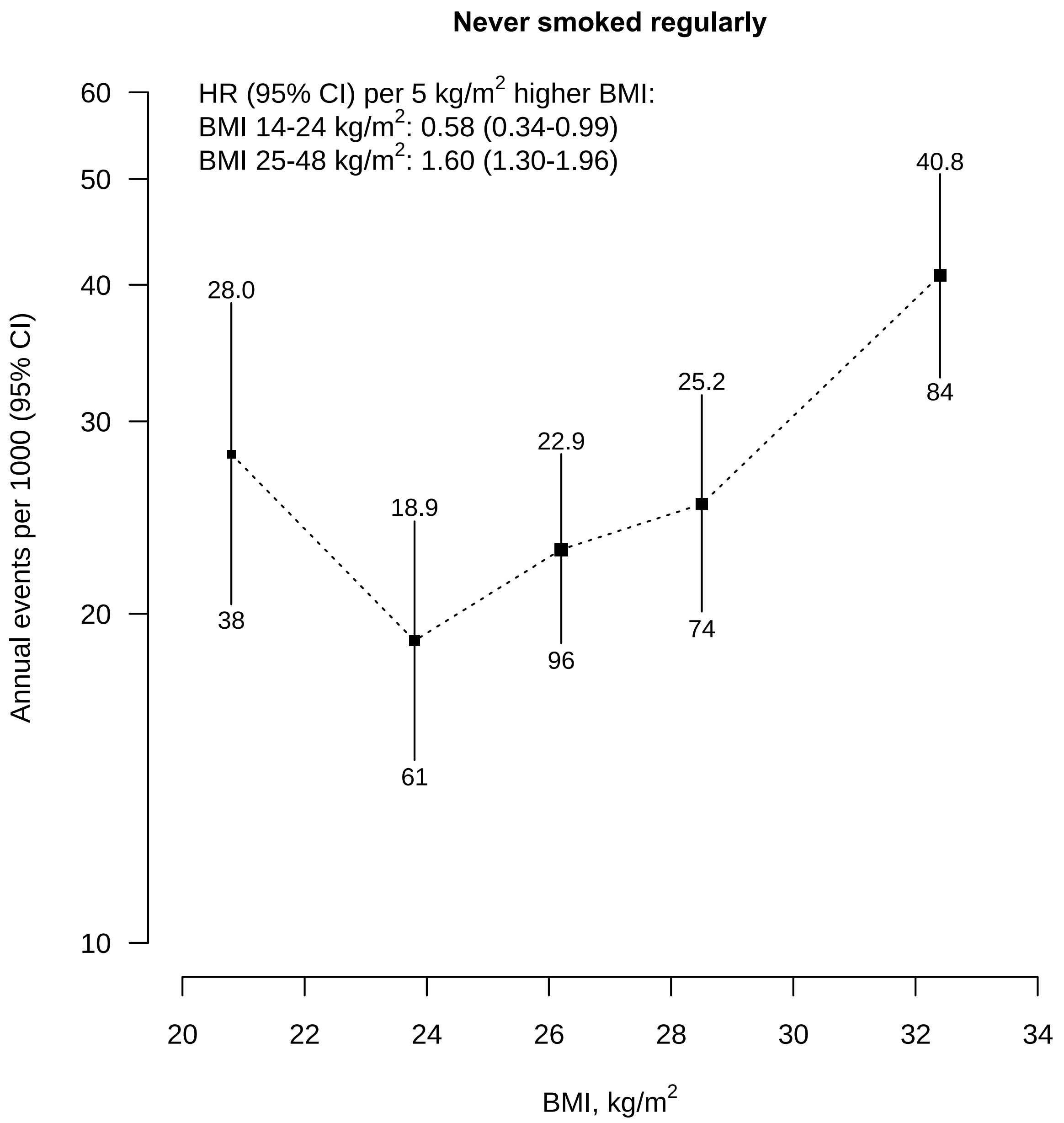

Figure S2. Incidence of major vascular events versus BMI in never smokers only (excluding the first 4 years of follow-up)

Hazard ratios adjust for age at risk, education and smoking. Conventions as per Figure 1.
